# A new species of *Helionothrips* from China (Thysanoptera, Panchaetothripinae)

**DOI:** 10.3897/zookeys.714.20644

**Published:** 2017-11-06

**Authors:** Zhaohong Wang, Xiaoli Tong

**Affiliations:** 1 Department of Entomology, College of Agriculture,; 2 South China Agricultural University, Guangzhou 510642, China

**Keywords:** *Helionothrips*, new species, Thripidae, thrips

## Abstract

*Helionothrips lushanensis* **sp. n.** is described from China. The new species is characterised by the head entirely dark brown, antennal segments I–VIII almost uniformly yellowish brown and III–IV strongly vasiform, metascutellum without produced posterior margins, and male has no pore plate on the abdominal sternites.

## Introduction

*Helionothrips* Bagnall, 1932, is one of the most species rich taxa in the subfamily Panchaetothripinae, currently comprises 28 described species in the world (ThripsWiki 2017). The species of the genus are mainly restricted in the Old World tropics and subtropics except for *H. funebris* (Hood, 1928) which is reported from South America. Of the described species, the majority of species (more than 80%) in the genus are known from the Asian region (Bhatti 1968; Wilson 1975; Zhang 1980; Kudô 1992, 1995; Wang 1993, 2002; Reyes 1994; Feng et al. 2007; Mirab-balou et al. 2017; ThripsWiki 2017). The review of the Panchaetothripinae species from China is available (Mirab-balou et al. 2017). In the present paper, a new species of the genus is added to the Chinese fauna.

## Materials and methods

The thrips were collected by beating vegetation over a white plastic tray using a stick, and then sorted and preserved in 90% alcohol. Examined specimens were mounted in Canada balsam using the method outlined by Zhang et al. (2006). Details of the morphological structures were examined with a ZEISS Imager A1 microscope; the photos were taken by a Photometrics CoolSNAP camera. All type specimens are deposited in the Insect Collection, South China Agricultural University (**SCAU**).

## Taxonomy

### Helionothrips lushanensis sp. n.

Taxon classificationAnimaliaThysanopteraThripidae

http://zoobank.org/7481BEA8-A3C4-4C92-A378-B723D6C04AF7

Figs 1–2 , 3–10

#### Material examined.

**Holotype** female (in SCAU): **CHINA**, Jiangxi province, Jiujiang City, Mt. Lushan (29°33'N, 115°59'E), collected from older leaves of *Ligustrum sinense* (Oleaceae), 9.xi.2015, leg. Xiaoli Tong.

#### Paratypes (in SCAU).

2 males from older leaves of *Ligustrum sinense* (Oleaceae), 4 males from older leaves of *Viburnum* sp. (Caprifoliaceae), 1 female from older leaves of *Rhododendron simiarum* (Ericaceae), all taken with holotype. Hunan province, Liuyang City, Daweishan National Forest Park (28°25'N, 114°06'E), 1 male from older leaves of *Rhododendron latoucheae* (Ericaceae), 15.viii.2016, leg. Zhaohong Wang.

#### Diagnosis.

Both sexes macropterous; body dark brown; head entirely dark brown; antennal segments I–VIII yellowish brown; fore wing brown with two pale bands. Head entirely reticulate without internal wrinkles within the reticules; antennae 8-segmented, segments III and IV strongly vasiform with forked sensoria, that on IV reach near the apex of V. Pronotum, meso- and metanotum completely reticulate, all lacking internal wrinkles within the reticles. Antecostal line on abdominal tergites III–VIII divided into broad arched sculpture with heavy anterior margin; tergite VIII with complete comb of microtrichia on posterior margin. Male similar to female in structure and colour but smaller; abdominal sternites without pore plates.

#### Description.

**Figures 1–2.**
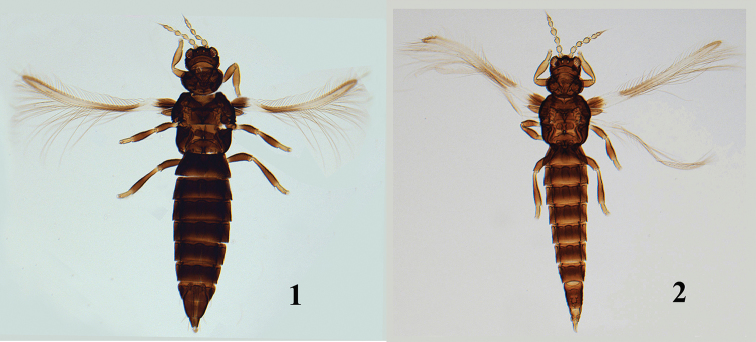
*Helionothrips lushanensis* sp. n. **1** female **2** male.

**Female** (*macropterous*): Body dark brown (Fig. 1), head entirely dark brown; antennal segments I–VIII yellowish brown or pale brown, I and VI slightly darker than other segments (Fig. 5). Fore legs yellowish brown, mid and hind legs dark brown except for the extremities of femora and tibiae yellowish brown; all tarsi yellow. Fore wing (Fig. 10) brown at base with white band sub-basally, brown at fork of veins and gradually fading apically, subapical pale band longer than sub-basal white band, ambient vein darker than surface of wing at apex, clavus dark brown.

**Figures 3–10.**
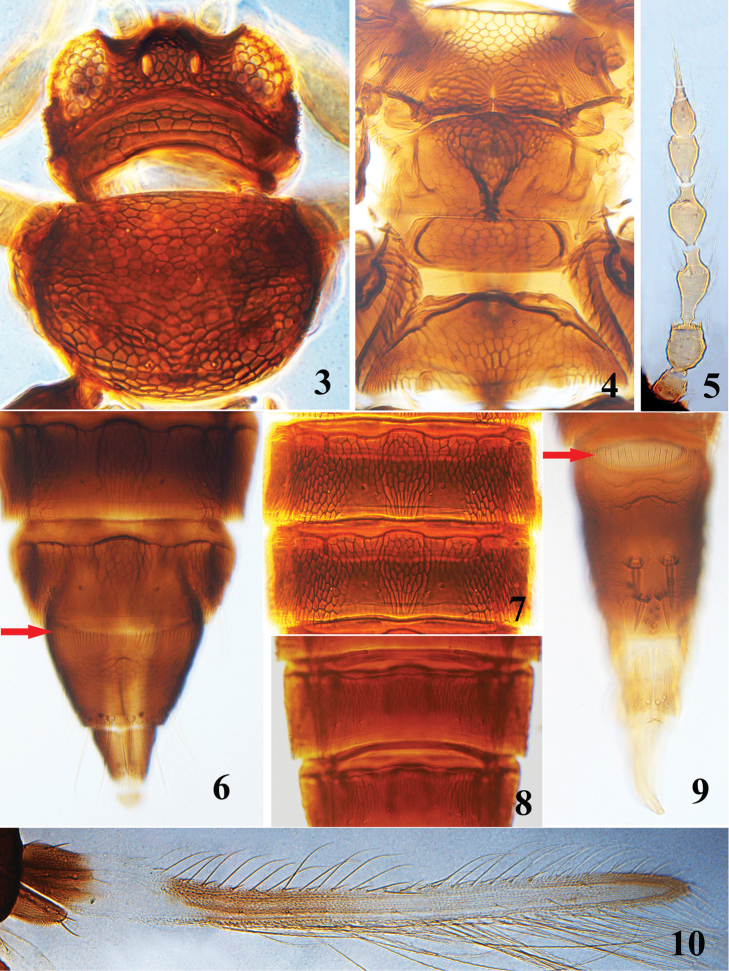
*Helionothrips lushanensis* sp. n. **3** head and pronotum **4** meso- and metanotum and abdominal tergite I **5** antenna **6** abdominal tergites VII–X **7** abdominal tergites IV–V **8** abdominal sternites VI–VII **9** abdominal tergites VIII–X of male **10** fore wing.

*Head* approximately twice as wide as long, sculptured with polygonal reticulations and without internal wrinkles within the reticules (Fig. 3); head with short, convex cheeks; occipital ridge strong and close to margin of eyes; occipital collar with numerous granules in posteromedian reticules; ocelli larger than any of the ommatidia and situated on the sides of an elevated hump. Antennae 8-segmented and stout, segments III and IV strongly vasiform with short apical neck, segment III longest with long pedicel, segment IV approximately 1.6 times as long as wide with very short basal pedicel (Fig. 5); segments III–IV with forked sensoria, that on IV reach near the apex of V; segments IV–VI with microtrichia rows on ventral surface, IV and V with 3 rows, VI with two rows. Mouth-cone rounded and moderately long, palps 2-segmented.

*Pronotum* wider than long, slightly ovoid (Fig. 3), with about 13–15 long and pale setae; pronotum, meso- and metanotum, metascutellum completely reticulate, all lacking internal wrinkles within the reticules (Fig. 4); metanotal median setae and campaniform sensilla arranging in a transverse line, median setae wide apart and far from anterior margin; metascutellum approximately three times as wide as long and posterior margins not produced. Fore wing first vein with approximately seven basal setae and two apical setae, second vein with 5–6 setae, clavus with four veinal and one discal setae; posterior marginal fringe cilia wavy. Legs with reticules, tarsi 1-segmented.

*Abdominal tergites* I and II covered with polygonal reticulations, III–VIII entirely reticulate except for the submedian smooth areas behind campaniform sensilla, with weak internal wrinkles within posterolateral reticules; antecostal line on tergites III–VIII divided into broad arched sculpture with heavy margin (Fig. 7); tergite VIII with complete comb of long microtrichia on posterior margin (Fig. 6); tergite IX with a pair of campaniform sensilla close to posterior margin and three pairs of stout and pointed setae along posterior margin, S1 and S2 setae subequal in length, but longer than S3 setae; tergite X smooth with median split complete. Abdominal sternites sculptured with longitudinal narrow reticules (Fig. 8).

*Measurements* (holotype female, in microns). Distended body length 1870. Head length (width) 100 (210); eye length (width) 70 (50). Pronotum length (width) 175 (250). Fore wing length 1040. Antennal segments I–VIII length (width) as follows: 26(25), 40(33), 65(29), 53(33), 40(25), 31(21), 10(9), 30(6).

**Male** (macropterous) (Fig. 2). Similar to female in structure and colour but smaller, abdomen more slender. Abdominal tergite VIII with complete comb of microtrichia on posterior margin (Fig. 9), tergite IX with two pairs of thorn-like setae, posterior pair shorter and closer to each other than anterior pair, a longitudinal cluster of 6–7 wart-like tubercles behind posterior pair (Fig. 9). Abdominal sternites without pore plates.

*Measurements* (paratype male, in microns). Distended body length 1570. Head length (width) 100 (175); eye length (width) 60 (40). Pronotum length (width) 140 (200). Fore wing length 840. Antennal segments I–VIII length (width) as follows: 20(22), 35(29), 58(27), 48(33), 33(24), 28(20), 8(9), 30(5).

#### Etymology.

The specific epithet is named after the type locality, Mt. Lushan, Jiujiang City, Jiangxi province, China.

#### Distribution.

China (Jiangxi, Hunan).

#### Remarks.

The new species is most similar to *H. errans* (Williams) in colour and structure, particularly in the pronotum lacking internal wrinkles within the reticules and abdominal tergite VIII having a complete comb on its posterior margin, but it can be distinguished from the latter by (1) head entirely dark brown (head anterior of fore ocellus yellow in *H. errans*); (2) antennal segments I–VIII almost uniformly yellowish brown (antennal segments I and III-V yellow, II and VI brown in *H. errans*); (3) antennal segments stouter, especially III and IV strongly vasiform, segment IV approximately 1.6 times as long as wide (in *H. errans*, the antennal segments slender, III and IV stalk-like, segment IV more than twice as long as wide); (4) metascutellum normal, lacking produced posterior margins (metascutellum with posterior margins produced in *H. errans*) and (5) male has no pore plate on the abdominal sternites (while the male has pore plates on sternites VII and VIII in *H. errans*).

## Supplementary Material

XML Treatment for Helionothrips lushanensis
